# Digital Health and Fiscal Credibility in Low‐ and Middle‐Income Countries: A Scoping Review of Practice‐Based Evidence

**DOI:** 10.1111/jep.70372

**Published:** 2026-02-03

**Authors:** Samuel Atiku, Olufisayo Olakotan

**Affiliations:** ^1^ Research Services Aston University Birmingham UK; ^2^ Digital Technology and Innovation University of Staffordshire Stoke‐on‐Trent UK; ^3^ Department of Neonatology, Women and Children's Directorate University Hospitals Leicester NHS Trust Leicester UK

**Keywords:** budget impact, digital health, fiscal credibility, fiscal rules, fiscal sustainability, low‐ and middle‐income countries (LMICs), public digital infrastructure, public financial management (PFM)

## Abstract

**Rationale:**

Digital health is widely promoted in low‐ and middle‐income countries (LMICs), yet investment is often constrained by fiscal rules and borrowing limits. Policymakers also struggle to explain how digital health improves fiscal sustainability and, by proxy, fiscal credibility, creating a gap between technical promise and investable policy narratives.

**Aims and Objectives:**

To synthesise practice‐based evidence on whether and how digital health initiatives in LMICs contribute to fiscal sustainability and signal fiscal credibility to governments, funders, investors, and to identify design and governance conditions that make these benefits investable under fiscal‐rule frameworks.

**Methods:**

A scoping review following PRISMA‐ScR analysed 45 studies (2010–2025) of LMIC digital health interventions that reported economic outcomes (e.g., cost‐effectiveness, cost savings, ROI, budget impact) and/or credibility proxies (e.g., integration with public financial management, budget absorption, stakeholder trust). Findings were synthesised thematically.

**Results:**

Evidence shows that digital platforms curb leakage and generate efficiency gains (e.g., reduced wastage, streamlined claims) and avert future costs through prevention and adherence, while integration with budgeting and public financial management improves predictability, transparency, and trust, thereby strengthening credibility signals. Yet benefits are frequently non‐cashable without managerial and policy reforms; costs and savings are misaligned across actors; and classifying digital outlays as recurrent restricts access to capital envelopes despite favourable economics. Investability improves when core platforms are treated as public digital infrastructure with clear depreciation and financing arrangements, when efficiency gains are translated into real budget space via gain‐sharing and performance‐based budgeting, and when reporting explicitly links digital performance to fiscal outcomes. These effects depend on foundational enablers, including infrastructure, workforce capacity, and sustained user engagement.

**Conclusion:**

When embedded in robust governance and public financial management, and framed as rule‐consistent investment, digital health can expand fiscal space, support compliance with fiscal rules, and strengthen fiscal credibility, helping LMICs reassure lenders and justify sustainable borrowing for health system strengthening.

## 1 Introduction

The expansion of digital health in Low‐ and Middle‐Income Countries (LMICs) presents a critical policy challenge. While technologies such as telehealth and electronic health records can modernise care and improve public health outcomes, the high upfront costs often require external borrowing. In fiscally constrained settings, LMICs must justify such borrowing under rules that prioritise long‐term, sustainable investment. This tension between digital innovation and fiscal accountability is the core of this inquiry [1, 2].

In many LMICs, fiscal frameworks, such as Nigeria's Fiscal Responsibility Act, require that public borrowing be directed to capital projects with lasting economic returns rather than short‐term operating costs. This is central to maintaining fiscal credibility, which is understood as confidence in the government's fiscal plans and its ability to meet obligations [3, 4]. Without credibility, countries face higher borrowing costs and weaker investor confidence [5].

Investors, including private creditors and international financial institutions, scrutinise indicators such as debt‐to‐GDP and debt‐servicing capacity. They are cautious about borrowing not tied to productive capital projects, which can raise perceived default risk and risk premia. Higher interest costs compress fiscal space for essential services and can trigger sovereign rating downgrades that further increase funding costs [6, 7, 8, 9].

Scholarship from global financial institutions and public finance experts increasingly frames digital health as a strategic, long‐term investment rather than a recurrent expense. Evidence suggests that although initial outlays can be high, long‐run savings are meaningful. A 2022 systematic review reported a generally favourable effect of digital health interventions on costs and health outcomes [10]. The World Bank argues that people‐centred, evidence‐based digital investments can help governments save up to 15 per cent of health costs through efficiency gains, reduced administrative burden, and better patient management [11].

Major international organisations now advocate an infrastructural approach. The World Health Organization's Global Initiative on Digital Health and recent World Bank work frame digital health systems as digital public infrastructure that underpins multiple use cases, analogous to roads or power grids. This emphasis on foundational platforms aligns with principles of strategic public investment and maximising spillovers [12, 13].

The International Monetary Fund IMF highlights that electronic health records and telemedicine can improve efficiency, resource use, and fiscal sustainability by generating reliable data and reducing waste [5, 11]. For LMICs, the challenge is justifying borrowing for digital health within accountability‐focused fiscal frameworks. This review addresses that gap by synthesising practice‐based evidence on how digital health contributes to fiscal sustainability and signals fiscal credibility, examining program design, financing, and integration, while also noting evidence gaps on budget impact, value distribution, and reinvestment of savings.

## 2 Methods

### 2.1 Research Question and Definitions

We conducted a scoping review following Arksey and O'Malley (2005) with Levac et al. (2010) enhancements, reported per PRISMA‐ScR [14, 15]. The question was: What practice‐based evidence exists on how digital health initiatives in LMICs contribute to fiscal sustainability?

For the purposes of this review, Low and Middle Income Countries were defined according to the World Bank classification system. This encompasses economies with a Gross National Income per capita falling within the low, lower middle, and upper middle income bands which are currently capped at $13,935 using the Atlas method [16, 17]. See Table 1.

**Table 1:** Definition of Income Categories (World Bank Atlas Method).

Income classification	GNI per capita range (USD)	Eligibility status
Low‐income	Up to $1,135	Included
Lower‐middle income	$1,136–$4,465	Included
Upper‐middle income	$4,466–$13,93E	Excluded
High‐income	$13,936 and above	Excluded

Fiscal sustainability denotes the capacity to deliver economic value over time (e.g., cost‐effectiveness, cost savings, return on investment) within local budgets. National fiscal credibility refers to stakeholder confidence in health financing and governance strengthened by demonstrable returns and efficient resource use. Practice‐based evidence included real‐world evaluations reporting financial outcomes or system‐level fiscal impacts across mHealth, eHealth, telemedicine, information systems, and supply‐chain or adherence technologies; “successful” interventions reported positive health or economic outcomes.

### 2.2 Search Strategy

We searched peer‐reviewed literature in PubMed, MEDLINE, Scopus, AJOL, Google Scholar and Web of Science Core Collection. The base search covered 2010 to 2025. Search strings combined two concept blocks:

1. Digital‐health modalities (tele/telehealth, mHealth/eHealth, electronic health record/health information system, disease surveillance, supply chain/logistics, including “supply NEAR/3 chain” in platforms that support proximity, drones, and digital adherence tools such as video‐observed therapy, 99DOTS, RapidSMS); AND
2. Economic/evaluation terms (cost‐effectiveness/utility/benefit, economic evaluation, budget impact, return on investment, cost saving, unit/program costs). Syntax was tailored per database.

To reduce noise, we searched the phrase “return on investment” (and avoided the acronym ROI) and used logistics (not logistic/logistical) to avoid logistic‐regression hits. We did not include LMIC/region terms in the search strings; eligibility by country income setting was applied during screening.

**Figure 1:**
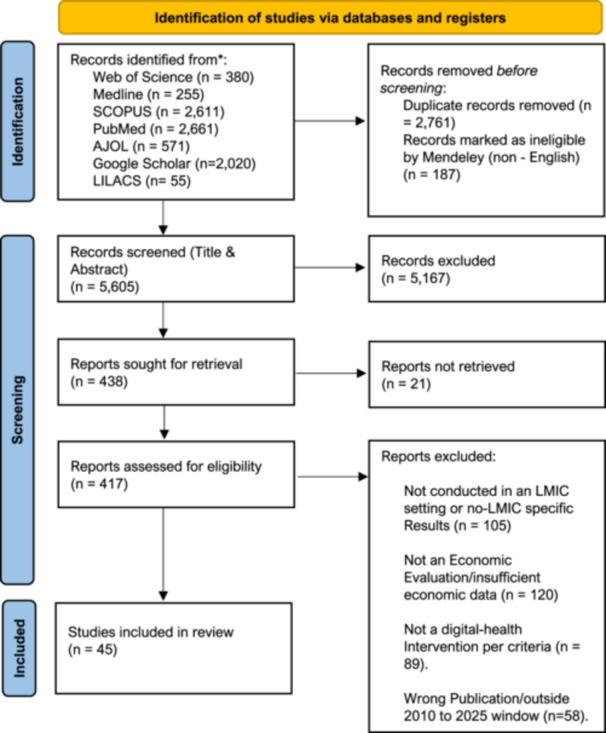
PRISMA flow diagram of study identification, screening, eligibility assessment, and inclusion.

To ensure comprehensive coverage and minimize geographical publication bias, we employed a multi‐phase search strategy targeting both global and region‐specific databases. Initial searches were conducted in January 2025 and updated in August 2025 across Web of Science (*n* = 380), MEDLINE (*n* = 255), Scopus (*n* = 2,611), and PubMed (*n* = 2,661). To further capture regionally indexed studies and reduce the skew toward high‐income country journals, the search was expanded in November 2025 to include African Journals Online (AJOL, *n* = 571), Google Scholar (*n* = 2,020), and a manual search of the LILACS database (*n* = 55). Full database‐specific search strings are provided in Appendix A. The screening and selection process for these records is detailed in the PRISMA flow diagram (Figure 1).

Eligibility Criteria and Study Selection: We included studies of any design (quantitative, qualitative, or mixed‐methods) that met the following criteria: (a) evaluated a digital health or eHealth intervention implemented in an LMIC (see Table 1); (b) reported economic or financial outcomes (such as cost‐effectiveness ratios, cost‐benefit or return on investment (ROI) measures, cost savings, budget impact, or economic proxy outcomes like reduced travel costs, efficiency gains), or provided evidence relevant to fiscal credibility (such as integration into national programs, policy adoption, or stakeholder trust outcomes); and (c) were published in English. Both experimental studies (e.g. randomised controlled trials, controlled before‐after studies) and observational or model‐based economic analyses (cost‐effectiveness analyses, budget impact models, benefit‐cost analyses) were eligible, as were relevant qualitative studies focusing on sustainability or financial aspects of digital health implementation.

We excluded articles that did not address financial outcomes (e.g. those focusing solely on clinical effectiveness without cost data), opinion pieces without empirical data, and studies from high‐income settings.

Studies on general health IT adoption or artificial intelligence were included only if they explicitly discussed economic sustainability or integration into health financing structures, to keep focus on our review question.

The selection process involved multiple reviewers for rigour. After removing duplicates, titles and abstracts were screened in Rayyan (a web‐based systematic review platform) for relevance. The two reviewers independently screened all abstracts against the inclusion criteria (with a third reviewer available to resolve any conflicts). Full‐text articles of potentially relevant studies were then retrieved and assessed by the two reviewers working in parallel. Disagreements on inclusion were resolved through discussion.

### 2.3 Data Extraction and Charting

Using a standardised form, we extracted author, year, country, intervention, setting, and key economic findings and relevant insights on sustainability or credibility. One reviewer extracted and a second verified. Extracted items were charted in summary tables; Table 2 summarises all 45 studies and Table 3 lists interventions and core economic outcomes.

**Table 2:** Summary characteristics and key cost and effectiveness findings of included digital health interventions in LMICs.

Citation	Intervention type	Setting	Key findings (cost & effectiveness)
[18]	Tele‐expertise (Tele‐ECG)	Cameroon	Reduced average patient costs by two‐thirds and was highly cost‐effective at Approximately US$44 per case managed.
[19]	Logistics management information system (cStock) & QI teams	Rwanda & Malawi	A qualitative study that found mobile‐enabled reporting and QI teams acted as enduring system assets to prevent fiscal leakage. No explicit cost figures.
[20]	Telemedicine (tele‐echocardiography)	Uganda	The cost was US29.48 per visit, estimated to decrease to US16.00 at optimal capacity. Patients cited cost‐ and time‐saving benefits.
[21]	Tele‐expertise (Tele‐EKG)	Côte d'Ivoire	The average patient cost of a traditional EKG was 3.8 times higher than a tele‐EKG. The program generated an average revenue per site of €5,529 over three years.
[22]	Digital screening and treatment (for viral hepatitis C)	Egypt	The program was projected to be cost‐saving by 2029, saving about $35 million in direct medical costs and averting 883,333 DALYs over 2018‐2030. N.B Although primarily a national screening/treatment programme, Egypt's HCV initiative deployed a web‐based national registration and appointment system and electronically uploaded screening results to a central database via computers/handheld devices, with automatic e‐referral to the nearest evaluation/treatment centre. We therefore classify it as a digital‐in‐health platform and include it in our corpus. In a model‐based economic evaluation, the programme was highly cost‐effective by 2021 and cost‐saving by 2029, with large projected health and cost gains over 2018–2030, illustrating prevention‐driven fiscal value when digital registries accelerate case‐finding and linkage to care.
[23]	Digital adherence technology (smart pillboxes)	Tanzania	A qualitative study that found DATs were perceived to reduce financial burden for patients and workload for health workers.
[24]	Logistics information system (eVIN)	India	Projected a return of INR 2.93 for every INR 1 invested in its sustainable phase. Value was driven by reduced vaccine wastage and stock optimization.
[25]	mHealth (mCARE)	Rural Bangladesh	Highly cost‐effective at a cost of $31 per DALY averted. The program had an 88% probability of being highly cost‐effective.
[26]	mHealth (SMS reminders)	Lagos, Nigeria	The return rate for child vaccinations was significantly higher (by 4.8%–6.0%). The incremental recurrent cost was US$7.90 per additional return case.
[27]	Digital adherence technology (99DOTS)	Uganda	A qualitative study that found the program was feasible and highly acceptable, but adherence declined over time, especially among people living with HIV.
[28]	mHealth (Kilkari)	India	Found to be highly cost‐effective, with a cost per life saved ranging from US 392 to US953 as the program matured.
[29]	Telemedicine (AI Screening)	China	AI telemedicine screening dominated no screening in rural settings. In urban settings, the ICUR was US 244, and the ICER was US2,567.
[30]	mHealth (ImTeCHO)	Gujarat, India	Highly cost‐effective at an incremental cost of US 74 per life − year saved or US5,057 per death averted in the per‐protocol analysis.
[31]	eLMIS and logistics management unit (LMU)	Tanzania	Upgrades reduced stock‐out odds by 49% and generated US$2.5 million in savings in the first year, defraying some investment costs.
[32]	Digital logistics (Drones)	Rwanda	A case study that found drone delivery reduced delivery times by an average of 79‐98 min and was associated with a 67% reduction in blood product expirations at 12 months.
[33]	Health system administration (e‐claims)	Ghana	The IBCR was 25.56 for the purchaser, but ‐35.20 for all providers. The overall health system IBCR was 90.06 when projected nationwide.
[34]	Digital adherence technology (99DOTS & VOT)	Multi‐country	Per‐person costs of 99DOTS were US98inBangladesh, US119 in the Philippines, and US$174 in Tanzania. VOT was more expensive but could be cost‐saving if donors covered fixed costs.
[35]	Digital logistics (Drones)	Ghana	Found drone delivery to be highly cost‐effective at US$58 per DALY averted and cost‐saving when replacing > 20% of ground transport.
[36]	mHealth (ReMIND)	Uttar Pradesh, India	From a health system perspective, the intervention had an incremental cost of US$205 per DALY averted. From a societal perspective, it was a cost‐saving intervention.
[37]	Telemedicine (Teleophthalmology)	Rural Southern India	From a health provider perspective, one‐off screening was cost‐effective at US$1,320 per QALY gained. Increasing to annual screening was not cost‐effective.
[38]	Digital adherence technology (VOT)	Moldova	Patients saved Approximately €25 (MDL 504) and 58 h of travel/wait time over a 4‐month period compared to in‐person DOT.
[39]	Public Health supply chain costing	Tanzania	A cross‐sectional costing study that found storage costs were the highest cost driver. The average unit cost per throughput was 22% at central MSD and 13% at zonal MSD. Although Ruhago et al. (2022) is a national supply‐chain costing rather than a before–after impact study, the analysis quantifies costs within Tanzania's digitally enabled supply chain—i.e., the Logistics Management Unit (LMU) operating an electronic LMIS (eLMIS) at national scale. Because the cost structure and performance indicators they report (e.g., storage as the dominant cost block, unit‐cost variation, order‐fill rates) are the outputs of an eLMIS/LMU platform, we classify this as digital‐in‐health and retain it in our corpus as a system‐level fiscal analysis of a discrete digital upgrade. For implementation details on the LMU/eLMIS upgrade and its performance effects, see Mwencha et al. (2017).
[40]	eCDSS	Rural Tanzania	The base‐case ICER was US$338 per 1% change in childbirth care quality. Overall quality improved by 23% but was not statistically significant.
[41]	Digital logistics (VDD)	Nigeria	Found the incremental cost for the VDD program to vaccinate one additional child was US$20.6.
[42]	Digital disease surveillance (eIDSR)	Sierra Leone	A national rollout of eIDSR required a total economic cost of approximately US 64,342, with projected annual direct operational costs of US14,091.
[43]	Digital adherence technology (99DOTS)	Uganda	The cost per treatment success was estimated at $355 in the trial‐specific scenario, falling to $59 with extended activities and $49 in a marginal clinic scenario.
[44]	mHealth (MOTECH)	Ghana	A cost‐effectiveness analysis found the cost per DALY averted fell sharply from US 174 in year one to US6.54 in year ten, with an average of US$20.94 over the decade.
[45]	Telemedicine (Tele‐Consultation Centre)	Ghana (Amansie‐West District)	Telemedicine was cost‐saving and cost‐effective with an Incremental Cost‐Effectiveness Ratio (ICER) of ‐US$351.75. Annualized total costs were US$227,006 for telemedicine compared to US$305,042 for conventional care 1.
[46]	Web‐based application (SA‐VigiApp)	South Africa	A cost minimization analysis found the web‐based application had the lowest cost of reporting (1.19 ZAR) compared to telephone (5.40 ZAR) or drop boxes (50.90 ZAR).
[47]	mHealth (Diabetes management)	Nigeria (South‐West)	70.6% of participants expressed willingness to pay for mobile phone‐based diabetic health services. Willingness was significantly associated with earning a higher income and educational status.
[48]	mHealth (SMS Reminders)	Nigeria (Lagos)	While 77% were willing to receive reminders, only 53% were willing to pay for them. The median amount participants were willing to pay was N10.00 (approx. US$0.06) per SMS.
[49]	mHealth (SMS Reminders)	Kenya (Nairobi)	The total implementation cost of the SMS intervention was US$99.08. It reduced failure‐to‐attend rates by 80% at 2 weeks (RR 0.2) and was deemed affordable relative to the clinical benefits.
[50]	mHealth (Mobile Link SMS/Voice)	Cambodia	The incremental cost was US$199 per person. The ICER was US$10,955 per DALY averted, exceeding the local threshold (US$1,671), indicating it was **not cost‐effective** at current effectiveness levels. Scaling up would cost approx. US$46 per person annually.
[51]	Electronic medical record system (EMRS)	Uganda	The total direct cost over 7 years was US$1,066,965 (approx. double the cost of a paper‐based system at US$544,159). However, costs decreased over time, and open‐source alternatives were estimated to be 20% cheaper (US$806,289).
[52]	mHealth (PIERS On the Move app)	India, Pakistan, Mozambique	Incremental cost per pregnancy was ~$12–16 across countries. Overall, the intervention was not cost‐effective due to low fidelity; however, for women receiving ≥ 8 contacts, it was highly cost‐effective (probability > 80%).
[53]	mHealth (Technology‐assisted peer therapy)	Pakistan	The intervention dominated standard care (WHO‐THP), being both more effective (0.005 incremental QALYs) and less costly (saving US$52 per patient). Real‐world delivery cost was estimated at US$24 per patient vs US$44 for standard care.
[54]	mHealth (CLIP Trial Cost Drivers)	Pakistan	A qualitative study identifying Out‐of‐Pocket (OOP) costs (transport, lost wages) and Program Implementation costs (infrastructure, training) as major cost drivers, informing the design of economic models for digital health.
[55]	Electronic Immunization Registries (EIRs)	Tanzania, Zambia	Total financial expenditures were US$4.2 M (Tanzania) and US$3.6 M (Zambia). Annualized cost per child was US$3.30–$3.81 in Tanzania and US$8.46 in Zambia. Hardware and deployment were the largest cost drivers.
[56]	Electronic Immunization Registries (EIRs)	Tanzania, Zambia	The intervention resulted in annual cost savings of US$10,236 per facility in Tanzania and US$628 per facility in Zambia, primarily driven by reductions in health worker time (efficiency gains).
[57]	AI‐powered clinical decision support	India	The program delivered a Social Return on Investment (SROI) ratio of 13:1 (social return of INR 34 M on investment of INR 2.6 M). Annual cost per child was INR 625. It reduced hospitalizations and out‐of‐pocket spending.
[58]	mHealth (SMS‐based medical education)	Vietnam	The total financial cost was US$49,552. The cost to achieve a 10% increase in knowledge was US$605 per clinician. A national scale‐up was estimated to cost US$196,446 over 10 years, deemed highly feasible.
[59]	mHealth (SMS Reminders)	Kenya	The intervention was highly cost‐effective. The cost per additional child correctly managed was US$0.50 under trial conditions and estimated at US$0.03 if scaled nationally due to economies of scale.
[60]	Telemedicine (multidisciplinary outpatient teleconsultations)	High‐complexity teaching hospital telemedicine programme, Cali, Colombia; patient‐perspective resource analysis (Apr–Dec 2020)	62,258 teleconsultations generated very large household cost savings through avoided travel. Total distance saved 4,514,903 km and time saved 132,886 h. Estimated transport cost savings were US$680,822 (private transport) and US$1,087,821 (public transport). Patients outside the main region saved on average ~21.2 h and US$149–157 per visit; air‐access areas saved ~US$362.9 per teleconsultation. Concludes telemedicine substantially reduces patient OOP and productivity losses and improves access in LMIC regions.
[61]	Telehealth/teleconsultation + tele‐training platform (UBS+ Digital)	Brazilian Unified Health System primary health units (PHUs) lacking on‐site physicians; multicenter prospective implementation (Mar–Nov 2023)	Teleconsultations resolved 85% of cases remotely with high satisfaction (NPS 97). Micro‐costing showed strong efficiency gains with scale: Total cost US$137/teleconsultation; Real (operational) cost US$85; Effective cost US$57; Optimized cost US$28 when capacity/visit length are optimized. Demonstrates a clear “glide path” where unit costs fall sharply as utilization improves.
[62]	Electronic medical record system (national EMR platform)	Mexican Social Security Institute (IMSS), Mexico; national EMR across primary care, outpatient, and hospitals (case study)	No formal CEA/ICER reported, but practice‐based evidence indicates primary‐care EMR improved efficiency of care delivery, human‐resource management, faster prescribing/leave certification, better data security, and may have reduced fraud/leakage. Hospital EMR modules showed lower coverage and weaker uptake due to workflow/infrastructure constraints. Overall fiscal value is framed as operational efficiency + fiduciary control, but with limited quantitative costing.

**Table 3:** Interventions and core economic outcomes across included studies.

Citation	Intervention/platform	Core economic outcome	Primary metric or economic result
Bediang et al. 2022	Tele‐expertise (Tele‐ECG)	Highly cost‐effective, patient cost‐saving	~US$44 per case managed; mean patient costs reduced by about two‐thirds
Chandani et al. 2017	cStock LMIS plus quality‐improvement teams	Economic impact not quantified	Qualitative evidence of reduced fiscal leakage and strengthened accountability
DeWyer et al. 2021	Tele‐echocardiography	Cost reduction with scale, patient cost/time savings	US$29.48 per visit, projected to ~US$16.00 at optimal capacity
Diby et al. 2021	Tele‐expertise (Tele‐EKG)	Cost‐saving to patients and revenue‐positive	Traditional EKG cost 3.8× higher; mean site revenue €5,529 over 3 years
Ezzat et al. 2023	Digital HCV screening registry and e‐referral	Highly cost‐effective then cost‐saving	Cost‐effective by 2021; cost‐saving by 2029; 883,333 DALYs averted
Gonçalves Tasca et al. 2024	Digital adherence technology (smart pillboxes)	Economic impact not quantified	Qualitative evidence of reduced financial burden and workload
Gurnani et al. 2022	eVIN logistics information system	Positive return on investment	INR 2.93 return per INR 1 invested in sustainable phase
Jo et al. 2019	mCARE messaging and registry	Highly cost‐effective	~US$31 per DALY averted
Kawakatsu et al. 2020	SMS vaccination reminders	Low incremental cost per outcome	US$7.90 per additional return visit
Kiwanuka et al. 2023	99DOTS digital adherence (qualitative)	Economic impact not quantified	Feasible and acceptable; adherence declined over time
LeFevre et al. 2023	Kilkari maternal messaging	Highly cost‐effective	US$392 to US$953 per life saved as programme matured
Liu et al. 2023	AI‐supported tele‐screening	Dominant or cost‐effective depending on setting	Dominates no screening in rural areas; urban ICUR US$244 and ICER US$2,567
Modi et al. 2020	ImTeCHO community mHealth	Highly cost‐effective	~US$74 per life‐year saved (per‐protocol)
Mwencha et al. 2017b	eLMIS plus logistics management unit	Cost‐saving	~US$2.5 M savings in first year; stock‐out odds reduced by 49%
Nisingizwe et al. 2022	Drone blood delivery	Efficiency gains not monetised	Delivery time reduced by ~79–98 min; expiries down by 67% at 12 months
Nonvignon et al. 2022	e‐claims administration	Net positive benefit‐cost, but asymmetric	Purchaser IBCR 25.56; provider IBCR − 35.20; projected system IBCR 90.06 nationwide
Nsengiyumva et al. 2024	99DOTS and video‐observed therapy (multi‐country costing)	Cost estimates, conditional value	99DOTS per‐person cost US$98 (Bangladesh), US$119 (Philippines), US$174 (Tanzania); VOT higher but potentially cost‐saving under donor‐covered fixed costs
Ospina‐Fadul et al. 2025	Drone delivery logistics	Highly cost‐effective; cost‐saving conditional	~US$58 per DALY averted; cost‐saving if replacing > 20% of ground transport
Prinja et al. 2018	ReMIND maternal and child mHealth	Cost‐effective and cost‐saving	US$205 per DALY averted (health system); cost‐saving societally
Rachapelle et al. 2013	Tele‐ophthalmology screening	Cost‐effective one‐off, not cost‐effective annual	~US$1,320 per QALY gained for one‐off screening; annual screening not cost‐effective
Ravenscroft et al. 2020	Video‐observed therapy for TB	Patient cost‐saving	~€25 and 58 h saved per patient over 4 months
Ruhago et al. 2022	National supply‐chain costing within eLMIS	Costing only	Storage largest cost driver; no ICER/ROI estimated
Saronga et al. 2017	eCDSS for childbirth care	Uncertain value due to weak effects	ICER US$338 per 1% change in care quality; quality gain not statistically significant
Sato et al. 2023	Vaccine direct delivery digital logistics	Low incremental cost per outcome	~US$20.6 per additional child vaccinated
Sloan et al. 2020	eIDSR disease surveillance	Costing and affordability only	Rollout ~US$64,342; annual operating costs ~US$14,091
Thompson et al. 2022	99DOTS costing and scenarios	Cost‐effective with scale	Cost per treatment success US$355 (trial), falling to US$59 and US$49 in scaled scenarios
Willcox et al. 2019	MOTECH maternal mHealth platform	Increasing cost‐effectiveness over time	Cost per DALY averted fell from US$174 (year 1) to ~US$6.54 (year 10)
Otsen and Agyei‐Baffour, 2016	Tele‐consultation centre	Cost‐saving	Negative ICER (−US$351.75); telemedicine annualised costs lower than conventional care
Adedeji‐Adenola and Nlooto, 2021	SA‐VigiApp web reporting	Cost‐minimising	Lowest cost per report at 1.19 ZAR versus phone or drop‐box routes
Olamoyegun et al. 2020	Diabetes mHealth (willingness‐to‐pay)	Economic outcome not quantified	70.6% willingness to pay; associated with income and education
Balogun et al. 2012	SMS reminders (willingness‐to‐pay)	Economic outcome not quantified	Median willingness to pay N10 ( ~ US$0.06) per SMS
Oramisi et al. 2019	SMS appointment reminders	Low cost and affordable	US$99.08 total cost; ~80% reduction in failure‐to‐attend
Avanceña et al. 2024	Mobile Link SMS and voice	Not cost‐effective	Incremental cost US$199 per person; ICER US$10,955 per DALY, above local threshold
Balugaba et al. 2020	Electronic medical record system	Higher direct cost than paper	~US$1.07 M over 7 years versus ~US$0.54 M paper; costs decreased over time, open‐source ~20% cheaper
Bone et al. 2021	PIERS On the Move app (multi‐country)	Not cost‐effective overall; cost‐effective conditional	~$12–16 per pregnancy; cost‐effective only for women with ≥ 8 contacts
Gibbs et al. 2025	Technology‐assisted peer therapy	Dominant	More effective and less costly than comparator; saving ~US$52 per patient
Khowaja et al. 2017	CLIP trial cost drivers (qualitative)	Economic impact not quantified	Identified major household and programme cost drivers for modelling
Mvundura et al. 2019	Electronic immunisation registries	Costing only	Financial spend US$4.2 M (Tanzania), US$3.6 M (Zambia); annualised US$3.30–3.81 per child Tanzania, US$8.46 Zambia
Mvundura et al. 2020	Electronic immunisation registries	Cost‐saving	Facility savings US$10,236 (Tanzania) and US$628 (Zambia) per year via staff time gains
Patil et al. 2023	AI‐powered clinical decision support	Positive social return on investment	SROI 13:1; reduced hospitalisations and out‐of‐pocket spending
Sabin et al. 2022	SMS medical education platform	Costing and feasibility only	US$605 per clinician per 10% knowledge gain; scale‐up feasible
Zurovac et al. 2011	SMS decision support for malaria	Highly cost‐effective	~US$0.50 per additional child correctly managed; ~US$0.03 at scale
Prada et al. 2024	Multidisciplinary telemedicine outpatient programme	Large patient cost‐saving	~US$0.68 M private and US$1.09 M public transport savings; major time and distance savings
Lamas et al. 2025	UBS + Digital teleconsultation and tele‐training	Strong scale‐related cost reduction	Unit cost fell from US$137 to US$57 at effective capacity, ~US$28 optimised
Humpage, 2010	National EMR platform (IMSS)	Economic impact not quantified	Practice‐based evidence of efficiency and fiduciary control, without formal ICER/ROI

### 2.4 Quality Appraisal

We used the Mixed Methods Appraisal Tool (MMAT) (2018) to appraise study quality. Instead of assigning summary scores, we performed an item‐level appraisal to provide a more nuanced understanding of the evidence base, in line with the MMAT authors' recommendations. Approximately 39 of studies out were rated high quality, with common weaknesses including small samples and incomplete cost scopes. The appraisal informed our synthesis by allowing us to weigh findings from higher‐quality studies more heavily and to temper our interpretations where methodological limitations were identified. No studies were excluded based on quality.

### 2.5 Synthesis of Results

We applied descriptive thematic synthesis. Two reviewers independently conducted first‐order coding of financial outcomes, followed by collaborative second‐order coding to group related codes and identify overarching themes. Four themes were derived, integrating quantitative estimates with contextual qualitative findings. Given heterogeneity, we undertook narrative synthesis rather than meta‐analysis, aligning with the scoping aim to map where and how digital health generates fiscal value and credibility.

## 3 Results

The summaries of the 45 articles included in the review are shown in Table 1. This section presents a narrative synthesis of our findings.

### 3.1 Overview of Study Design

We identified 45 eligible studies spanning 25 Low‐ and Middle‐Income Countries (LMICs). Five studies employed a multi‐country design (Bone et al. 2021; Chandani et al. 2017; Mvundura et al. 2019; Nsengiyumva et al. 2024), yielding a total of 52 country instances. Geographic distribution was uneven: India and Tanzania were the most frequent settings (*n* = 7 each), followed by Uganda, Nigeria, and Ghana (*n* = 4 each). A smaller cluster (Kenya, Rwanda, Bangladesh, Zambia) contributed two studies each, while the remaining 15 countries were represented singly.

#### 3.1.1 Study Designs and Economic Focus

To avoid double‐counting, studies were assigned a single primary analytic design. The majority (*n* = 27) were economic evaluations or costing analyses, reflecting a predominant focus on fiscal credibility. The remainder comprised quasi‐experimental/implementation evaluations (*n* = 11), qualitative or mixed‐methods studies (*n* = 4), and randomized controlled trials (*n* = 3). While diverse, this distribution suggests the current evidence base is weighted heavily toward pragmatic economic modelling and observational data rather than experimental efficacy trials.

### 3.2 Key Themes From Practice‐Based Evidence on Digital Health in LMICs

#### 3.2.1 Theme 1: Direct Fiscal Savings and Operational Efficiency: Costing, Cash Flow, and Economic Fluidity

Across diverse contexts, the fiscal credibility promise of digital health is real, yet who captures the savings, when they appear, and whether they take the form of cash or cost avoidance depends on design choices and programme maturity.

Cost‐effectiveness and ROI typically improve over time as digital tools displace inefficient cost bases rather than merely computerise tasks. India's eVIN shows this maturation: modest early returns with capital and start‐up costs, stronger returns with a wider product mix, and compelling steady‐state performance once recurrent costs dominate [24]. Brazil's UBS+Digital project illustrates a clear utilisation “glide path”: unit costs fell from US$137 per teleconsultation to US$57 at effective capacity and about US$28 under optimized scheduling and throughput [61].

Savings stem largely from improved stock management rather than headline IT spend, underscoring that value lies in reducing logistics waste through better information and control [24]. A parallel pattern appears in Ghana's MOTECH, where cost per DALY averted falls sharply from year one to year ten, with gains tied to concurrent provider data capture and client messaging [44]. Similar trajectories are observed in large mHealth platforms such as Kilkari, where cost per life saved drops as the programme matures and reaches national scale [28].

Operational streamlining yields fiscal value when systems cut waste, delay and rework, not just paperwork. Tanzania's eLMIS and LMU upgrades reduced stock‐out odds and generated multi‐million‐dollar savings in the first year by preventing emergency procurement and expired inventory [63]. However, companion supply‐chain costing reveals these gains are not automatic; storage costs remained high, implying that digital transparency must be paired with inventory governance to convert information into cashable savings [39]. Nigeria's Vaccine Direct Delivery and related digital distribution initiatives similarly point to low marginal delivery costs per additional child vaccinated, indicating scalability when logistics are redesigned around the tool rather than layered onto legacy processes [41].

Time‐critical aerial logistics strengthens this argument. Rwanda's drone delivery reduced times by over an hour and sharply cut expiries, implying immediate avoidance of urgent road trips and wastage write‐offs [32]. Ghana's analysis of aerial logistics shows potential per‐dose cost savings once network effects and avoided wastage are counted [35].

Administrative automation also shifts who benefits. Ghana's e‐claims platform is economically superior to paper, but gains are asymmetric: purchasers accrue high benefit–cost ratios from leakage control, while providers face compliance costs. Efficiency dividends accrue first to the payer unless sharing mechanisms support provider adoption [33]. A similar “low‐cost rail” logic appears in South Africa's pharmacovigilance web‐app, where unit reporting costs are far lower than analogue channels, suggesting that administrative digitisation can generate near‐immediate recurrent savings when workflows are redesigned around the platform [46].

Patient affordability gains are immediate and material in tele‐expertise models, often with facility revenues that support sustainability. In Côte d'Ivoire and Cameroon, tele‐ECG cut out‐of‐pocket costs significantly and avoided millions of travel kilometres [18, 21]. Uganda's tele‐echocardiography shows comparable household savings and a glide path toward lower per‐visit costs [20], At district scale in Ghana, tele‐consultation centres proved cost‐saving relative to conventional referral care [45]. Similar dividends appear in Latin America: Colombia's multidisciplinary telemedicine programme generated large productivity savings, avoiding 4.5 million km of travel and saving roughly US$1.77 M in transport costs [60].

Large digital registries concentrate value in prevention and efficiency at scale. Egypt's hepatitis C initiative, supported by a national registry, was projected to become cost‐saving within the decade via reduced future treatment liabilities [22]. Electronic Immunisation Registries in Tanzania and Zambia highlight a more granular mechanism. Although start up expenditures are substantial, annualised per child costs are low and facility level savings accrue through reduced health worker time and better visit efficiency [55, 56].

Finally, digitisation can surface costs if scope expands without redesign. Uganda's EMR case documented higher direct costs than paper systems initially, though unit costs fell over time. This suggests procurement choices and interoperability matter as much as the decision to digitise [51]. Fiscal value emerges not from software possession, but from selecting affordable architectures to remove redundant work.

Taken together, value capture is design‐dependent. Stock intelligence and surveillance concentrate value in reduced wastage and low recurring administrative costs [24, 42]. Tele‐expertise shifts value to households and peripheral facilities, while e‐claims centralises it at the purchaser unless incentives are rebalanced [18, 21, 33]. Timing differs: waste‐ and travel‐reducing tools such as drones and tele‐ECG yield immediate savings, whereas information platforms such as eLMIS and eIDSR need a glide path before savings exceed capital and change‐management costs [32, 35, 39, 42]. Countries that pair national roll‐out with mechanisms that convert operational wins into budget relief are best placed to make information gains become cash gains, for example by redeploying storage savings, tightening claims leakage, and ring‐fencing wastage reductions.

#### 3.2.2 Theme 2. Preventative Economics and Averted Costs

Digital tools protect health and public finances when they move systems from reactive to proactive care, avert complications, and target high‐burden risks. A national HCV programme in Egypt shows the arc clearly. Upfront spend rises, followed by sharp declines in disease and costs. The model projects 883,333 DALYs averted and direct medical savings by 2030, with the programme becoming cost saving by 2029 [22].

Digital adherence technologies for tuberculosis offer potential efficiencies through economies of scale. Although the 99DOTS intervention in Uganda showed neutral clinical effectiveness, economic analysis indicated it is fiscally viable: modelling projected the cost per treatment success would drop to $59 when scaled up [43]. This suggests digital tools can become cost‐effective by reducing patient support costs, even without immediate clinical superiority. Multicountry costing shows that while costs remain moderate, qualitative work warns that prevention value erodes when adherence technologies lose intensity among high‐risk groups [27, 34].

Maternal–newborn messaging shows similar prevention value. In India, Kilkari was highly cost‐effective at national scale, saving an estimated 13,842 lives at a cost of US$392–US$953 per life saved [28]. In Bangladesh, adding SMS and home‐visit prompts to a digital registry averted DALYs at roughly US$31 each [25]. Additional platforms reinforce this: ImTeCHO in Gujarat and ReMIND in Uttar Pradesh proved cost‐effective or cost‐saving by preventing complications and reducing household spending [30, 36]. Conversely, PIERS On the Move became cost‐effective only where contact intensity was adequate, showing that prevention returns depend heavily on delivery fidelity [52].

Tele‐expertise reallocates costs away from households and avoids unnecessary treatment. In Uganda, remote cardiology triage excluded disease for many, keeping per‐visit costs low [20]. In Côte d'Ivoire and Cameroon, tele‐ECG networks cut patient prices and eliminated large travel burdens while maintaining favourable cost‐effectiveness [18, 21]. Prevention gains also appear in screening: teleophthalmology in rural India was cost‐effective for early detection, though annual screening was not, highlighting the importance of periodicity (Rachapelle et al. 2013). Similarly, AI‐supported screening in China dominated no‐screening scenarios in rural settings, showing prevention value when technology expands reach (Liu et al. 2023).

Low‐cost behavioural nudges compound these gains by preventing lapses. Kenya trials of SMS reminders for malaria management found very low costs per additional child correctly treated [59]. For immunisation, reminder systems in Nigeria and Kenya reduced non‐attendance and improved follow‐up at minimal incremental cost, pointing to scalable prevention value in routine services [26, 48, 49].

Taken together, the strongest fiscal case appears where digital tools preempt costly events. Screening and treatment platforms collapse future disease burden; adherence technologies avert expensive failures; and tele‐expertise strips out overtreatment. The consistent pattern is prevention converted into credible economic value when information is timely, actions are automated, and incentives align across programmes [18, 20, 21, 22, 25, 26, 28, 29, 30, 34, 36, 37, 43].

#### 3.2.3 Theme 3. Governance, Stakeholder Trust, and Fiscal Cohesion

Digital health platforms gain fiscal credibility and long‐term viability not merely by introducing technology, but by integrating into broader systems of governance and shared accountability. Tanzania's national LMU and eLMIS illustrate this lesson: central coordination and data visibility improved planning and stock outcomes, producing efficiency gains. However, short‐term management improvements were uneven, underlining the need for sustained stewardship to convert information into performance [39, 63].

Beyond top‐down governance, successful digital interventions also foster multi‐level collaboration. In Malawi and Rwanda, formal quality‐improvement teams linked community, facility and district actors to reinforce resupply procedures around an SMS‐enabled logistics system, aligning roles, supervision and problem‐solving so that digital rails were backed by routine accountability [19]. Similar complementarities appear in decision support and learning platforms. In rural Tanzania, eCDSS improved childbirth care quality but effects were not statistically significant, suggesting that weak integration into supervision and clinical routines limits downstream value [40]. Vietnam's SMS based medical education platform was financially feasible at scale, yet its value proposition depended on national coordination for curriculum integration and sustained professional uptake [58].

Collaboration extends to national ownership and fiscal policy, key to signaling credibility. In India, the eVIN program scaled to all 731 districts with the government requiring states to absorb recurrent costs. This strategic move paired ROI with budgetary integration, reassuring funders of durability beyond pilots [24]. Similarly, the Kilkari maternal messaging program transitioned to government stewardship by 2019, marking stable policy commitment [28]. Large registry‐based programmes show similar effects: Egypt's hepatitis C initiative utilized a national digital platform to generate a prevention‐driven fiscal case, strengthening confidence in execution capacity [22].

Digital administration can also tighten fiduciary control. Ghana's e‐claims system improved error detection and leakage control, demonstrating economic advantage for the payer while highlighting the need to share dividends so providers adopt without bearing net losses [33]. South Africa's SA VigiApp provides a related governance signal by sharply lowering unit reporting costs compared with analogue channels, strengthening confidence in the regulatory chain [46]. In Sierra Leone, the eIDSR rollout coupled visible capital investment with low recurrent costs to support transparent decision‐making and partner confidence [42].

Procurement and architecture choices further shape credibility. Uganda's EMR experience showed higher direct costs than paper systems initially, indicating that digital value is sensitive to decisions about vendor models and interoperability [51]. In the tuberculosis space, qualitative studies of 99DOTS show declining engagement over time, implying that governance must include ongoing user support to sustain the fiscal logic of prevention [27, 43]. Smart pillboxes in Tanzania were perceived as beneficial, but evidence remains qualitative, reinforcing that trust and routines are prerequisites for measurable cost‐effectiveness [23]. Evidence from Mexico's IMSS national EMR similarly highlights workflow fit: primary‐care EMRs improved efficiency and fraud control, whereas hospital modules struggled with complexity, underscoring that credible savings depend on participatory design [62]

Taken together, fiscal credibility grows when countries pair digital platforms with clear stewardship, budget absorption, and multi‐level teams that translate data into practice. Where these conditions hold, information gains become performance gains. Where they do not, digital tools risk exposing inefficiencies or generating uneven benefits that weaken trust [19, 22, 23, 24, 27, 28, 33, 39, 40, 42, 51, 58, 63].

#### 3.2.4 Theme 4. Overcoming Hurdles in the Digital Landscape

Digital health delivers only when basic enablers, patient engagement, and system capacity move together. Where phones, power, and connectivity are fragile, even well‐designed tools falter. Qualitative work on tuberculosis adherence in Tanzania shows that feasibility depends on reliable handsets, networks, and electricity. Users reported battery depletion, weak signals, and glitches that caused confusion and unplanned clinic visits, adding hidden costs for households [23]. A parallel constraint appears in modelling AI‐supported teleophthalmology in China: without policy attention to referral compliance and workforce training, rural–urban gaps risk widening as urban settings adopt more quickly [29].

Keeping people engaged over time is equally difficult. In Uganda, self‐reported dosing through 99DOTS declined monthly despite reminders. Drops were more pronounced among people living with HIV, reflecting motivation fatigue and the labour required to sustain support actions [27]. Stigma amplifies these frictions when devices reveal diagnosis, pushing users to avoid digital features even when access exists [23].

Platforms also need system capacity behind them. Rwanda's RapidSMS improved service use only in districts that coupled messaging with training and equipment; messaging alone did not shift outcomes, underscoring that information must be matched with supplies to convert signals into action [64]. Tanzania costing work echoes this: digitising logistics increased visibility but could not deliver savings without complementary inventory governance, reminding decision‐makers that data without stewardship rarely changes performance [39]. Similarly, a Latin‐American EMR case showed incomplete rollout where infrastructure and budgets lagged behind the platform [62].

Methodological gaps further complicate the picture. Several studies rely on models rather than prospective tracking and often omit granular costs such as patient time, AI maintenance, and device replacement. These gaps bias benefit–cost assessments, reinforcing the need for studies combining user outcomes with full economic accounting [29, 39].

Pragmatism works. Pair digital rails with enablement, as seen in RapidSMS districts [64]. Match adherence tools to user context regarding charging and connectivity [23]. Update reimbursement for AI‐supported screening so providers break even [29]. Under these conditions, technical promise becomes durable performance [23, 29, 39].

Taken together, fiscal credibility depends on last‐mile conditions rather than technology alone. Across studies, infrastructure constraints, uneven literacy, and engagement drops often convert nominally low‐cost tools into higher hidden costs. Furthermore, platforms deliver muted returns where supply chains and supervision are not strengthened in parallel. The cumulative lesson is that digital investments become fiscally persuasive only when paired with reliable infrastructure, maintenance plans, equity safeguards, and governance that aligns incentives. Without these complements, programmes risk fragile returns; with them, technical promise converts into durable utilization and savings that partners can trust [23, 27, 29, 39].

## 4 Discussion

**Figure 2:**
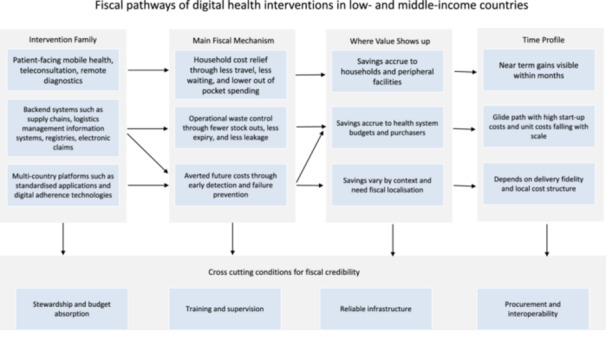
Fiscal pathways of digital health interventions in low‐ and Middle‐income countries.

The practice‐based evidence across 25 countries indicates that digital health has shifted from a clinical add‐on to a structural route for allocative efficiency. Fiscal credibility, however, does not follow automatically; it emerges through a temporal and causal sequence summarized in Figure 2.

Economic value depends on the intervention class, the fiscal mechanism, and program maturity. Across the corpus, liquidity was generated through reduced supply‐chain leakage, lower patient out‐of‐pocket spending, and averted future treatment liabilities. Yet these gains were repeatedly conditioned by maturity. In most settings, costs were front‐loaded, while the savings required for credibility became persuasive only once utilization increased and routine processes stabilised [24, 28, 44, 61]. This timing implies that financing must treat digital systems as long‐term infrastructure, with repayment horizons aligned to delayed efficiency dividends, rather than as short pilots expected to deliver immediate cash savings.

A further implication concerns the trade‐off between ease of implementation and fiscal impact. The evidence distinguishes lower‐friction, patient‐facing tools from structural backend reforms. Patient‐facing interventions, including tele‐consultation and SMS reminders, are typically easier to deploy because they use existing consumer phones and avoid major infrastructure build‐outs. They provide quick, visible equity gains by reducing travel time and opportunity costs for households [18, 45, 60]. Related evidence in LMICs confirms similar reductions in non‐medical costs and access barriers [10]. However, the state's budgetary benefit is indirect unless payment rules and referral pathways are adjusted to capture downstream efficiencies. Conversely, backend platforms, such as electronic logistics management systems, are more demanding as they require hardware procurement, connectivity, workforce training, and sustained managerial oversight. Yet these are the interventions most consistently linked to tangible fiscal release for governments through reductions in wastage, stock‐outs, and emergency procurement [24, 39, 63]. A plausible sequencing logic follows: governments may start with patient‐facing tools to secure rapid access gains, while recognizing that long‐term solvency is more likely to stem from the heavier lift of backend digitisation.

The review also addresses multinational viability. Software architectures travel well, but economic performance remains context‐bound. Multi‐country costings of identical platforms showed notable unit‐cost variation, driven by local labor markets, connectivity prices, and service density [34]. The cost of supporting digital adherence in Tanzania, for example, diverged sharply from Bangladesh because baseline infrastructure differed [34]. Multi‐country trials also imply that cost‐effectiveness depends more on delivery fidelity than on code alone [52]. Multinational interventions are therefore feasible and may share development costs, yet they require local fiscal planning. Supervision, hosting, and training budgets must reflect the maturity of each digital ecosystem rather than assuming stable cost performance across heterogeneous settings [10, 65].

Governance and last‐mile conditions ultimately determine whether digital value becomes fiscal credibility. Where stewardship, budget absorption, and reliable infrastructure were present, information gains translated into performance gains [24, 28, 42]. Where power, connectivity, or data‐use routines were weak, digitization could expose inefficiency rather than reduce it [23, 27, 39]. The credibility signal to investors thus comes less from the technology than from public commitment to the accompanying reforms that allow it to function, including procurement discipline, inventory management, and workforce supervision. Future research should move beyond short‐horizon models to prospective, longitudinal budget‐impact studies that test whether projected efficiency gains become audited savings that are retained and reinvested. This is particularly important given the recurring omission of granular costs such as device maintenance and patient time [29, 39, 65].

### 4.1 Implications for Future Research

Evidence should extend beyond short‐term cost‐effectiveness to system‐level budget impacts, with longitudinal and budget‐impact studies verifying whether efficiency gains translate into audited savings that are retained and reinvested. Fiscal credibility should be measured with practical indicators such as recurrent budget absorption, shifts in financing, and public financial management metrics, supported by insights from finance officials. Studies need to map who pays and who benefits, testing incentive‐compatible arrangements where purchaser savings and provider costs are misaligned, as suggested by the e‐claims experience in Ghana [33]. Comprehensive perspectives are needed in economic analyses, incorporating life‐cycle technology costs, training, and patient time, with transparent price years and sensitivity analyses.

At the macro level, simulation models should embed digital health within national expenditure frameworks to estimate effects on fiscal space, coverage, financial risk protection, and equity. Implementation science approaches such as CFIR and NASSS can help link determinants to realised returns, moving from descriptive lessons to predictive indicators of success.

### 4.2 Strengths and Limitations

This review is among the first to synthesize practice‐based economic evidence of digital health in LMICs, bridging cost‐effectiveness research with insights on system‐level sustainability. By incorporating diverse study designs and outcomes, it captures both quantitative metrics and qualitative context, offering a nuanced view of how digital health performs financially in real‐world settings beyond what single‐method studies provide.

A key strength is the focus on practice‐based evidence, including national programs and government‐led initiatives, rather than only controlled research environments. This enhances the policy relevance of the findings and demonstrates feasibility and impact at scale, as shown in multi‐country and national rollouts, rather than being limited to small pilot projects.

The studies in this review varied widely in interventions and outcomes, preventing meta‐analysis; thus, conclusions are thematic rather than quantitative. Reliance on peer‐reviewed literature introduces publication bias, as positive results are more likely to be reported than failures. Despite including regional indices (AJOL, LILACS) to counter database bias, the evidence remains geographically skewed toward Anglophone Africa and India, with limited representation from Latin America, the Middle East, or Francophone regions. Additionally, our strict application of World Bank income thresholds excluded potentially relevant evidence from graduated high‐income economies (e.g., Chile, Uruguay). Finally, fiscal credibility was inferred from proxies like budget integration rather than direct measurement, rendering the synthesis exploratory. Future research should address these gaps by expanding geographic coverage and developing direct fiscal metrics.

## 5 Conclusion

In low‐ and middle‐income countries, digital health initiatives are emerging as strategic investments that can improve health outcomes while strengthening the financial sustainability and credibility of health systems. This scoping review of 45 studies shows that well‐designed digital interventions, including mHealth applications, telemedicine services, logistics management systems, and digital adherence technologies, often deliver measurable economic benefits. These benefits include cost savings, favourable cost‐effectiveness ratios, and operational efficiencies that help make better use of limited resources. By reducing waste, preventing costly health events, and streamlining service delivery, digital health demonstrates clear fiscal value when effectively integrated into health systems.

The impact, however, goes beyond financial metrics. Digital platforms that enhance transparency and accountability can strengthen fiscal credibility by showing donors and investors tangible evidence of responsible innovation and measurable results. Governments that embed such tools into routine systems signal reliability and stewardship, which builds confidence and attracts further investment. Yet these gains depend on supportive environments, including good governance, policy reforms, reliable infrastructure, and strong stakeholder engagement. Without these, the benefits of digital health may not be fully realised. Taken together, the evidence suggests that digital health in LMICs can be highly cost‐effective, sometimes cost‐saving, and capable of contributing to a cycle of improved health outcomes and stronger financial stewardship.

## Conflicts of Interest

The authors declare no conflicts of interest.

## Supporting information

Appendix.

## Data Availability

Data sharing not applicable to this article as no datasets were generated or analysed during the current study.

## Appendix A Database Search Results and Search Strings

**Table:**

Database	Result	Search strings
Web of Science	380	(telemedicine OR telehealth OR mhealth OR ehealth OR “electronic health record*“ OR “health information system*“ OR “disease surveillance” OR (“supply NEAR/3 chain”) OR logistics OR drone* OR “video observed therap*“ OR *SMS) AND (“cost‐effect*“ OR (“cost NEAR/1 benefit*“) OR (“cost NEAR/1 utility”) OR “economic evaluat*“ OR “budget impact” OR “return on investment” OR “cost saving*“ OR “unit cost*“ OR “program cost*“)
Medline	255	(telemedicine OR telehealth OR mhealth OR ehealth OR “electronic health record*“ OR “health information system*“ OR “disease surveillance” OR (“supply NEAR/3 chain”) OR logistics OR drone* OR “video observed therap*“ OR *SMS) AND (“cost‐effect*“ OR (“cost NEAR/1 benefit*“) OR (“cost NEAR/1 utility”) OR “economic evaluat*“ OR “budget impact” OR “return on investment” OR “cost saving*“ OR “unit cost*“ OR “program cost*“)
SCOPUS	2,611	(telemedicine OR telehealth OR mhealth OR ehealth OR “electronic health record*“ OR “health information system*“ OR “disease surveillance” OR (supply W/3 chain) OR logistics OR drone* OR “video observed therap*“ OR *SMS) W/5 (“cost‐effect*“ OR (cost W/1 benefit*) OR (cost W/1 utility) OR “economic evaluat*“ OR “budget impact” OR (“return W/1 investment”) OR “cost saving*“ OR “unit cost*“ OR “program cost*“)
Pubmed	2,661	(telemedicine OR telehealth OR mhealth OR ehealth OR “electronic health records” OR “health information systems” OR “disease surveillance” OR “electronic logistics management information system” OR “video observed therapy” OR *SMS OR telecardiology OR “tele‐ECG” OR drone OR drones) AND (“cost‐effectiveness” OR “cost‐utility” OR “cost‐benefit” OR “economic evaluation” OR “budget impact” OR “return on investment”)
AJOL	571	mHealth, “mobile health”, telemedicine, Digital health, eHealth, “cost‐effectiveness”, “economic evaluation”, “cost‐benefit”
Google Scholar	2,020	allintitle: (telehealth OR telemedicine OR mHealth OR eHealth OR “digital health” OR EHR OR “health information system” OR “disease surveillance” OR “supply chain” OR logistics OR drones OR 99DOTS OR RapidSMS) (“cost‐effectiveness” OR “economic evaluation” OR “budget impact” OR “return on investment” OR “cost savings” OR “unit cost” OR “program cost”)
LILACS	55	mHealth, “mobile health”, telemedicine, Digital health, eHealth, “cost‐effectiveness”, “economic evaluation”, “cost‐benefit”

## Appendix B Intervention Classes and Associated Fiscal and Economic Outcomes

**Table:**

Intervention class	Key economic findings (ranges and typical values)
Tele‐expertise and telemedicine	Provider or programme costs: Observed unit costs ranged from about US$29.48 per tele‐echocardiography visit in Uganda, projected to fall to US$16.00 with adequate utilisation, to US$137 per teleconsultation in Brazil, falling to US$57 at effective capacity and about US$28 when optimised (DeWyer et al. 2021; Lamas et al. 2025). Cost‐effectiveness: Tele‐ECG in Cameroon was highly cost‐effective at about US$44 per case managed, and tele‐consultation centres in Ghana were cost‐saving with a negative ICER of about US$351.75 (Bediang et al. 2022; Otsen and Agyei‐Baffour, 2016). Teleophthalmology was cost‐effective for one‐off screening at about US$1,320 per QALY, but not cost‐effective if repeated annually (Rachapelle et al. 2013). AI tele‐screening dominated no screening in rural China and remained cost‐effective in urban settings, with an ICER of about US$2,567 and an ICUR of about US$244 (Liu et al. 2023). Household savings: Patient out‐of‐pocket and travel savings were substantial where measured, including about a two‐thirds reduction in patient costs in Cameroon, an exam about 3.8 times cheaper in Côte d'Ivoire, and large avoided travel and time costs in Colombia averaging about US$149 to US$157 per visit in remote regions and about US$363 in air‐access areas, with total transport savings of more than US$1.7 million (Bediang et al. 2022; Diby et al. 2021; Prada et al. 2024).
Digital logistics and supply‐chain platforms (drones, eVIN, eLMIS and LMU, VDD)	Return on investment and savings: India's eVIN projected INR 2.93 returned for every INR 1 invested once the recurrent phase was established (Gurnani et al. 2022). Tanzania's eLMIS and LMU upgrade generated about US$2.5 million in savings in the first year and reduced stock‐out odds by about 49 per cent (Mwencha et al. 2017b). Cost per outcome: Nigeria's Vaccine Direct Delivery had an incremental cost of about US$20.6 per additional child vaccinated (Sato et al. 2023). Drone logistics: Rwanda's drone programme reduced delivery times by about 79 to 98 min and reduced blood expirations by about 67 per cent, showing efficiency gains though not monetised (Nisingizwe et al. 2022). Ghana's modelled drone analysis found about US$58 per DALY averted, with cost‐saving possible when drones replace more than 20 per cent of ground transport (Ospina‐Fadul et al. 2025). Cost structure evidence: National supply‐chain costing in Tanzania showed storage as the dominant cost block and throughput unit costs varying across levels, indicating that savings depend on governance and fulfilment quality, not digitisation alone (Ruhago et al. 2022).
Digital adherence technologies (99DOTS, video‐observed therapy, smart pillboxes)	Cost per treatment success: Trial‐specific cost per treatment success for 99DOTS was about US$355, but fell to about US$49 to US$59 under realistic scale or marginal clinic scenarios, indicating strong scale sensitivity (Thompson et al. 2022). Per‐person programme cost: Multi‐country costing estimated 99DOTS costs at about US$98 per patient in Bangladesh, US$119 in the Philippines, and US$174 in Tanzania. Video‐observed therapy had higher programme costs but could become cost‐saving if fixed costs are externally covered (Nsengiyumva et al. 2024). Household savings: In Moldova, video‐observed therapy reduced patient costs by about 25 euros and 58 h over 4 months relative to in‐person DOT (Ravenscroft et al. 2020). Qualitative fiscal signals: Smart pillboxes and 99DOTS feasibility studies reported perceived reductions in burden and workload, though without quantified savings (Gonçalves Tasca et al. 2024; Kiwanuka et al. 2023).
mHealth beneficiary messaging and decision support (maternal and child health, reminders, education, willingness‐to‐pay studies)	Cost per DALY or life saved: Kilkari's cost per life saved ranged from about US$392 to US$953 as the platform matured nationally (LeFevre et al. 2023). mCARE in Bangladesh cost about US$31 per DALY averted (Jo et al. 2019). ImTeCHO in India cost about US$74 per life‐year saved (Modi et al. 2020). ReMIND cost about US$205 per DALY averted to the health system and was cost‐saving societally (Prinja et al. 2018). MOTECH's cost per DALY averted improved steeply with scale, from about US$174 in year one to about US$6.54 by year ten (Willcox et al. 2019). Low‐cost nudges: SMS vaccination reminders in Nigeria cost about US$7.90 per additional return visit (Kawakatsu et al. 2020). SMS appointment reminders in Kenya cost about US$99 in total, reducing failure‐to‐attend by about 80 per cent (Oramisi et al. 2019). Malaria SMS decision support in Kenya cost about US$0.50 per additional child correctly managed, projected to fall to about US$0.03 at national scale (Zurovac et al. 2011). Economic evidence not quantified: Diabetes and immunisation willingness‐to‐pay studies reported willingness to pay but no cost‐effectiveness estimates (Olamoyegun et al. 2020; Balogun et al. 2012). Clinical‐effectiveness SMS studies without costing were also present (Lester et al. 2010; Pop‐Eleches et al. 2011). Professional education: SMS medical education cost about US$605 per clinician per ten per cent knowledge gain (Sabin et al. 2022).
Administrative systems, registries, EMRs, surveillance, and AI clinical decision support	Claims and insurance administration: Ghana's e‐claims system produced a strong purchaser benefit‐cost ratio, with an IBCR of about 25.56, but negative ratios for providers of about minus 35.20 unless savings are shared. National scale projection yielded net positive system value of about 90.06 (Nonvignon et al. 2022). Surveillance: Sierra Leone's eIDSR rollout cost about US$64,342 upfront, with about US$14,091 in annual operating costs, supporting affordability with a capital plus low‐recurrent profile (Sloan et al. 2020). Digital reporting: South Africa's SA‐VigiApp reduced reporting costs to about 1.19 ZAR per report versus higher analogue costs (Adedeji‐Adenola and Nlooto, 2021). EMRs: Uganda's EMR cost about US$1.07 million over 7 years, about double a paper system, although costs declined over time and open‐source alternatives were estimated cheaper (Balugaba et al. 2020). Mexico's national EMR case reported efficiency and fiduciary gains without formal ICERs (Humpage, 2010). Immunisation registries: EIR investments were sizeable at US$4.2 million in Tanzania and US$3.6 million in Zambia, but annualised costs were low at about US$3.30 to US$3.81 per child in Tanzania and about US$8.46 in Zambia. Facility savings accrued through reduced staff time, about US$10,236 per facility in Tanzania and about US$628 per facility in Zambia (Mvundura et al. 2019; Mvundura et al. 2020). AI clinical decision support: India's AI‐CDSS showed a high social return, with an SROI of about 13 to 1 and reductions in hospitalisation and household costs (Patil et al. 2023). National screening registry: Egypt's digital HCV registry and e‐referral platform was modelled to become cost‐saving by 2029, averting about 883,333 DALYs and saving about US$35 million in direct medical costs (Ezzat et al. 2023).
